# Comparative study of transbronchial cryobiopsy and transbronchial biopsy for diagnostic yield in peripheral pulmonary lesions

**DOI:** 10.1080/07853890.2026.2613456

**Published:** 2026-01-08

**Authors:** Hao-Chun Chang, Ching-Kai Lin, Lun‑Che Chen, Ling‑Kai Chang, Shun‑Mao Yang, Li-Ta Keng, Chong‑Jen Yu

**Affiliations:** ^a^ Division of Pulmonary and Critical Care Medicine, Department of Internal Medicine, National Taiwan University Hsinchu Branch, Biomedical Park Hospital, Hsinchu County, Taiwan; ^b^ Division of Pulmonary and Critical Care Medicine, Department of Internal Medicine, National Taiwan University Hospital, Taipei, Taiwan; ^c^ Division of Pulmonary and Critical Care Medicine, Department of Internal Medicine, National Taiwan University Cancer Center, Taipei City, Taiwan; ^d^ Division of Chest Surgery, Department of Surgery, National Taiwan University Hsinchu Branch, Biomedical Park Hospital, Hsinchu County, Taiwan; ^e^ Division of Pulmonary and Critical Care Medicine, Department of Internal Medicine, National Taiwan University Hsinchu Branch, Hsinchu Hospital, Hsinchu City, Taiwan

**Keywords:** Transbronchial biopsy, transbronchial cryobiopsy, augmented fluoroscope, peripheral pulmonary lesions

## Abstract

**Background:**

Transbronchial cryobiopsy (TBCB) is a minimally invasive technique that yields larger specimens than conventional transbronchial forceps biopsies (TBFB) and demonstrates superior diagnostic rates for interstitial lung diseases. However, the efficacy of TBCB compared to TBFB in evaluating peripheral pulmonary lesions (PPLs) is not well established. This study aims to examine the diagnostic performance of TBCB relative to TBFB in PPLs.

**Material and methods:**

Between May 2021 and December 2023, patients with PPLs were enrolled and underwent TBFB followed by TBCB. These procedures were performed either in a hybrid operating room (HOR) or a standard bronchoscopy room without fluoroscopy. The study compared histopathology diagnostic yield between the two methods.

**Results:**

The study included 84 patients. The median lesion size was 37 mm (interquartile range: 26, 54), with 16 lesions (19.0%) measuring less than 2.0 cm. Among the participants, 44 (52.4%) were diagnosed with lung cancer, and 28 (33.3%) had infectious diseases. TBCB yielded significantly larger tissue samples [60 mm^3^ (range: 30, 144) vs. 4 mm^3^ (range: 2, 6), *p* < 0.001] and higher diagnostic yields (94.0% vs. 77.1%, *p* < 0.001) than TBFB. The higher diagnostic yield for TBCB were consistent in both the bronchoscopic room (97.2% vs. 77.8%, *p* = 0.008) and HOR (91.5% vs. 76.6%, *p* = 0.033). The incidence of ≥ grade 3 bleeding was 7.1%.

**Conclusion:**

TBCB significantly improves the diagnostic yield for PPLs, irrespective of fluoroscopic guidance, and is effective for both malignant and benign lesions. Furthermore, it is associated with minimal complications, affirming its safety and efficacy as a diagnostic procedure.

**Highlights**
1. TBCB consistently provided a higher pathological yield compared to TBFB, independent of lesion size, use of fluoroscopy, or the nature of the pathology (benign or malignant)
2. TBCB yielded larger tissue sample and had high successful rates for NGS testing.
3. Combination of an ultrathin bronchoscope, augmented fluoroscopy, ROSE, and TBCB can lead to high diagnostic yields.

## Introduction

In recent years, the detection of peripheral pulmonary lesions (PPLs) has become more frequent due to the increasing use of chest computed tomography (CT) scans, particularly in lung cancer screening [1,2]. These lesions, which can be indicative of a range of diseases including lung cancer, lung metastases of other cancers, lymphoma, benign tumors, tuberculosis, and various infections or inflammatory processes, often present a diagnostic challenge. Historically, CT-guided transthoracic needle biopsy has been the gold standard for diagnosing PPLs, offering a superior diagnostic yield [3,4]. However, its application is limited by the risk of complications such as pneumothorax and pulmonary hemorrhage [4,5]. These complications have raised concerns about the safety and feasibility of CT-guided biopsy as a long-term diagnostic tool, prompting the exploration of safer, minimally invasive alternatives.

In response, various bronchoscopic modalities have emerged as promising techniques for diagnosing PPLs. These include thin or ultrathin bronchoscopes, radial endobronchial ultrasound (r-EBUS), bronchoscopic guide sheath (GS), virtual bronchoscopic navigation (VBN), electromagnetic navigation bronchoscopy (ENB), cone beam CT (CBCT), and robotic bronchoscopy [6–8]. While these methods significantly reduce complications compared to CT-guided biopsy, they often yield smaller and less adequate tissue samples, affecting diagnostic accuracy [9,10]. A primary challenge with many bronchoscopic techniques is the size and quality of the biopsy specimens. For instance, transbronchial forceps biopsy (TBFB) often produces small, sometimes crushed samples that may complicate diagnosis and impede molecular testing [11–13].

A promising alternative to traditional bronchoscopic techniques is transbronchial cryobiopsy (TBCB). This procedure utilizes a cryoprobe capable of extracting larger tissue samples with better preservation of cellular architecture and fewer artifacts compared to forceps biopsies. The cryoprobe operates by freezing the tissue, facilitating a 360° lateral biopsy that yields larger and more intact specimens [14,15]. Transbronchial lung cryobiopsy (TBLC) has proven particularly valuable in the evaluation of interstitial lung diseases (ILDs) and has attracted increasing interest as a potential tool for sampling PPLs [16–18]. The integration of r-EBUS to guide cryobiopsy has further enhanced the safety and precision of the procedure. By utilizing r-EBUS to identify and avoid blood vessels at the biopsy site, clinicians can minimize the risk of complications such as bleeding [19]. However, the effectiveness of TBCB in diagnosing PPLs had not been established previously [20]. Therefore, this study was conducted to explore the potential role of TBCB in PPLs.

## Materials and methods

### Study patients

This study was conducted at the Biomedical Park Hospital of National Taiwan University (NTUH) Hsinchu Branch, a regional hospital in northern Taiwan. Patients included in the study were those with solid PPLs on CT scans, showing a positive bronchus sign, and who agreed to undergo self-paid TBCB from May 2021 to December 2023, since cryoprobes are not covered by Taiwan’s National Health Insurance program. All patients would receive TBFB as well. The study was performed in accordance with the principles stated in the Declaration of Helsinki and approved by the Institutional Review Board of NTUH (202501147RIN) on March 4^th^, 2025. The protocol and the request for the waiver of informed consent for retrospective collection of medical records was also approved by the Research Ethics Committee of the NTUH.

### Bronchoscopic procedure

Patients underwent bronchoscopic procedures either in a hybrid operation room (HOR) equipped with augmented fluoroscope (AF) and cone bean computed tomography (CBCT) or in a standard bronchofiberscopic (BFS) room without fluoroscopic assistance. In general, patients with smaller and more peripheral (further than the fourth generation of bronchial segments) lesion would be designated in a HOR. Those undergoing procedures in the HOR recieved general anesthesia with endotracheal intubation for airway management, while patients in the BFS room were administered intravenous sedation, supported by a high-flow nasal cannula. The ultrathin 3.0-mm bronchoscope, with a 1.7-mm working channel (BF-MP290F; Olympus), was used in majority, while thin scopes, with a 4.2-mm width and 2.0-mm working channels (BF-P290; Olympus), standard 4.8-mm bronchoscope, with a 2.0-mm working channel (BF-Q290; Olympus), and therapeutic scopes, with a 5.9-mm width and 3.0-mm working channels (BF-1TQ290; Olympus), were the other options.

For those who underwent the procedures in the HOR, CBCT was performed once the anesthesia was administered and the lesion was marked and overlaid ton the AF screen. After navigating the bronchoscope to the lesion segment localized by the CT scan, a 1.4-mm diameter r-EBUS probe (UM-S20-17S; Olympus) was advanced through the working channel toward the lesion. If the target lesion was identified by r-EBUS, it would be marked on the AF image. In cases where a concentric image was not obtained, intra-procedural CBCT was employed to explore better access routes. Once the target was set, TBFB was executed at the marked site using biopsy forceps suitable for the scope’s working channel size—either 2.0-mm (FB-231D; Olympus) or 1.5-mm (FB-433D; Olympus). Following TBFB, TBCB was also performed on the marked area using a cryoprobe (available in sizes 1.1, 1.7, or 2.4 mm; Erbe).

As for patient receiving the procedures in the BFS room, r-EBUS was also utilized to locate the lesion. Once the target lesion was found by r-EBUS, the distance from the segment orifice was measured. TBFB was then performed with the assistance of a guide sheath (GS) (K201 or K203; Olympus; depending on the working channel size of the scope used). After sampling, the GS was withdrawn from the working channel, and a cryoprobe was inserted to conduct TBCB. TBFB was taken at least four times for each patient, and TBCB was executed at least once. Rapid on-site cytological evaluation (ROSE) was also conducted for both type of specimen to increase the diagnostic yield. Notably, neither VBN nor ENB were employed in these procedures.

If bleeding occurred after the biopsies and could not be controlled by bronchoscopic wedge compression, epinephrine irrigation was applied. If bleeding persisted after two administration of 1 mg epinephrine with two minutes of wedge compression each, a balloon catheter was then deployed.

### Evaluation parameters

The primary endpoint of the study was the histopathological diagnostic yield of TBFB and TBCB. Additional analyses included diagnostic yield, procedure time, and the frequency of complications, all stratified by the location where the procedures were performed. Bleeding was classified into four grades, as previous reported [21].

A positive diagnostic yield was defined by pathology reports identifying malignant or benign neoplasms, granulomatous inflammation, organizing pneumonia, or the presence of fungi or acid-fast positive microorganisms. Conversely, a non-diagnostic yield comprised findings of normal lung tissue, non-specific fibrosis, or chronic inflammation. Patients were followed for a minimum of six months or until final diagnoses were established, based on the combination of pathological evidence, microbiological culture results, radiological imaging, and treatment outcomes. Notably, if pneumonia or lung abscess was the clinical diagnosis and the lesion completely resolved after antimicrobial treatment during follow-up, inflammation from the initial pathology report would also be considered as a positive diagnosis.

### Statistical analysis

Categorical variables were compared using the McNemar’s test, with McNemar’s exact test applied as necessary. Continuous variables were expressed as mean ± standard deviation (SD) and were analyzed using paired t-test. For continuous variables failed to pass a normality test, values were presented as median and quartiles, and comparisons were made using the Wilcoxon signed rank test. A p-value of less than 0.05 was considered statistically significant. All statistical analyses were conducted using SAS version 9.4 (SAS Institute, Cary, NC, USA).

## Results

**Figure 1.**
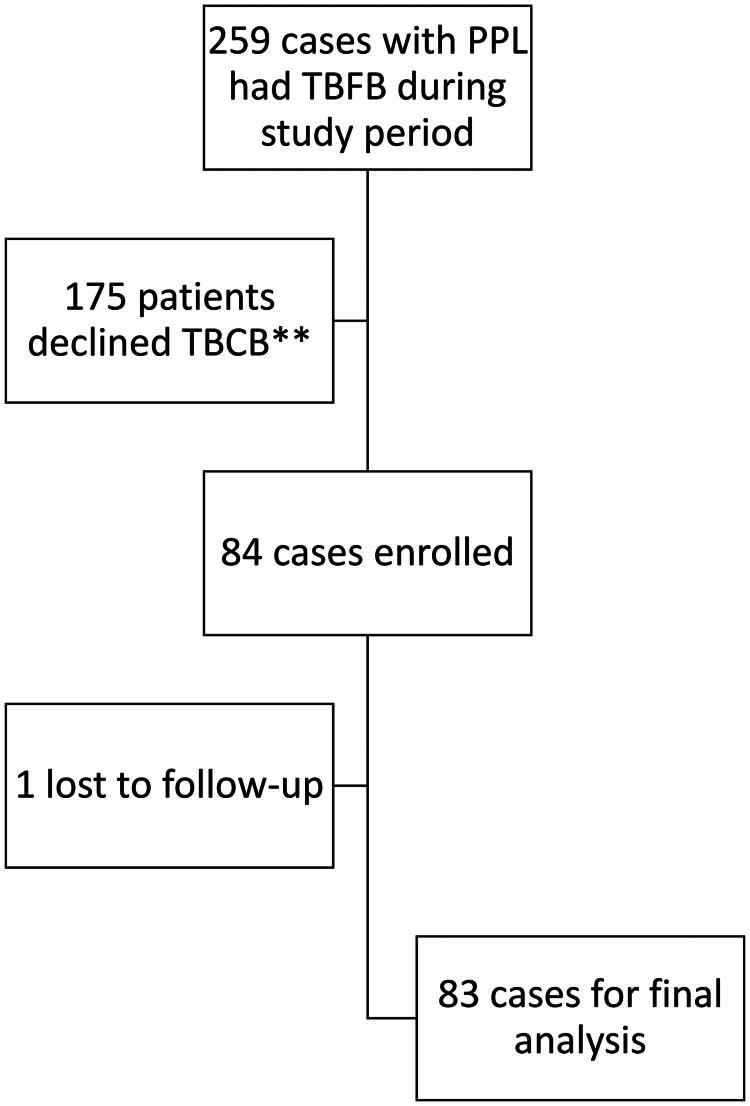
Flowchart of patient enrollment. * PPL: peripheral pulmonary lesion, TBFB: transbronchial forceps biopsy, TBCB: transbronchial cryobiopsy ** These patients declined to receive TBCB due to unwilling to pay for the procedure fee, since it was not reimbursed by Taiwan’s National Health Insurance

A total of 84 patients were enrolled in the study, as shown in Figure 1. Baseline characteristics of the patients, their lesions, and bronchoscopic findings are detailed in Table 1. The majority of the lesions measured ≥ 20 mm in diameter (81.0%), with a median size of 37 mm (interquartile range: 26, 54). Approximately half the procedures (57.1%) were performed in the HOR. The thin scope (P scope) and ultrathin scope (MP scope) were the most commonly utilized, accounting for 26.2% and 36.9% of uses, respectively. Most patients (82.1%) recieved TBCB with a 1.1 mm cryoprobe. Concentric lesions could be found by r-EBUS for most patients (86.9%). There were seven instances of complications (8.3%), comprising one pneumothorax and six grade 3 bleeding. No grade 4 bleeding occurred in the study groups. The final diagnoses of the patients are shown in Table 2. The majority of the diagnoses were lung cancer (52.4%), especially adenocarcinoma (39.3%).

**Table 1.** Characteristics of the patients and their lesions.

	Patients (*n* = 84)
Age (mean ± SD)	65.9 ± 12.1
Sex (Male)	
Male	49 (58.3%)
Female	35 (41.7%)
Smoker	36 (42.9%)
Lesion size (cm) (median, quartile)	3.7 (2.6, 5.4)
≥2 cm	68 (81.0%)
<2 cm	16 (19.0%)
Augmented fluoroscopy	48 (57.1%)
Lesion lobe	
Right lung	45 (53.6%)
Right upper lobe	16 (19.0%)
Right middle lobe	8 (9.5%)
Right lower lobe	21 (25.0%)
Left lung	39 (46.4%)
Left upper division	15 (17.9%)
Left lingual lobe	9 (10.7%)
Left lower lobe	15 (17.9%)
Bronchoscope type	
BF-1TQ290	19 (22.6%)
BF-Q290	12 (14.3%)
BF-P290	22 (26.2%)
BF-MP290	31 (36.9%)
Cryoprobe type	
2.4	2 (2.4%)
1.7	13 (15.5%)
1.1	69 (82.1%)
EBUS image	
Concentric	73 (86.9%)
Eccentric	11 (13.1%)
Procedure time (minutes)	64.9 ± 24.3
Complications	7 (8.3%)

* EBUS: endobronchial ultrasound.

**Table 2.** Final diagnoses of the patients.

Final Diagnosis	Patients (*n* = 84)
Lung cancer	44 (52.4%)
Adenocarcinoma	33 (75.0%)
Squamous cell carcinoma	1 (2.3%)
Small cell carcinoma	5 (11.4%)
Other lung cancer	5 (11.4%)
Benign	39 (46.4%)
Mycobacterial infection	6 (15.4%)
Bacterial pneumonia	11 (28.2%)
Lung abscess	5 (12.8%)
Fungal lung	6 (15.4%)
Organizing pneumonia	7 (17.9%)
Others	4 (10.3%)
Lost to follow-up without a final diagnosis	1 (1.2%)

Detailed procedural data are presented in Table 3. The average number of biopsies taken was significantly higher for TBFB at 7.5 compared to 3.5 for TBCB (*p* < 0.001). Additionally, the sample size obtained *via* TBCB was substantially larger than that *via* TBFB [TBCB vs. TBFB: 60 (30, 144) mm³ vs. 4 (2, 6) mm³, *p* < 0.001]. TBCB also demonstrated a higher pathology diagnostic yield than TBFB (94.0% vs. 77.1%, *p* < 0.001).

**Table 3.** Comparison of the results of cryobiopsy and forceps biopsy.

	Cryobiopsy (*n* = 84)	Forceps biopsy (*n* = 84)	P value
Number of biopsies	3.5 ± 2.6	7.5 ± 2.8	<0.001
Sample size (mm^3^) (median, quartile)	60 (30, 144)	4 (2, 6)	<0.001
Pathology yield rate	94.0% (78/83*)	77.1% (64/83*)	<0.001

* There was one case lost to follow-up without a final diagnosis.

In a subgroup analysis, sample sizes were consistently larger in TBCB across various lesion sizes and types. For lesions smaller than 20 mm, TBCB yielded 30 (23, 63) mm³ compared to TBFB’s 2 (1, 4) mm³ (*p* < 0.001). For lesions 20 mm or larger, the sizes were 66 (30, 153) mm³ for TBCB and 4 (2, 8) mm³ for TBFB (*p* < 0.001). The pathology diagnostic yield for TBCB remained higher regardless of lesion size—for lesions under 20 mm, the diagnostic yield was 93.8% versus 62.5% for TBFB (*p* = 0.031); for lesions 20 mm or larger, 94.0% versus 80.6% (*p* = 0.011). For malignant lesions, TBCB showed a numerically higher diagnostic yield of 88.6% compared to TBFB’s 75.0%, though this difference was not statistically significant (*p* = 0.054). For benign lesions, the yield was 100% for TBCB compared to 79.5% for TBFB (*p* = 0.004).

There were no significant differences in procedure time based on lesion size (lesions < 20 mm: 61.3 ± 21.9 min vs. lesions ≥ 20 mm: 65.8 ± 24.7 min, *p* = 0.488). Similarly, complication rates did not differ significantly between smaller and larger lesions (12.5% vs. 7.4%, *p* = 0.799). These details are further expanded in Tables 4 and 5.

**Table 4.** Results stratified by lesion size.

	Lesions < 2.0 cm (*n* = 16)			Lesions ≥ 2.0 cm (*n* = 68)			P value
Lesion size (cm) (median, quartile)	1.5 (1.2, 1.7)			3.9 (3.5, 5.7)			<0.001
Lung cancer	3 (18.8%)			41 (60.3%)			0.003
BFS type							
MP scope	12 (75.0%)			19 (27.9%)			<0.001
P scope	3 (18.8%)			19 (27.9%)			
Others	1 (6.2%)			30 (44.1%)			
Cryoprobe type							
1.1	14 (87.5%)			55 (90.9%)			0.835
Others	2 (12.5%)			13 (19.1%)			
AF usage	15 (93.8%)			33 (48.5%)			0.001
Procedure time	61.3 ± 21.9			65.8 ± 24.7			0.488
Complication rate	2 (12.5%)			5 (7.4%)			0.799
	TBCB	TBFB	P value	TBCB	TBFB	P value	
Sample size (mm^3^) (median, quartile)	30 (23, 63)	2 (1, 4)	<0.001	66 (30, 153)	4 (2, 8)	<0.001	
Pathology yield rate	93.8% (15/16)	62.5% (10/16)	0.031	94.0% (63/67*)	80.6% (54/67*)	0.011	

* There was one case lost to follow-up without a final diagnosis.

** BFS: bronchofibroscope, AF: augmented fluoroscope, TBCB: transbronchial cryobiopsy, TBFB: transbronchial forceps biopsy.

**Table 5.** Results stratified by procedure location and diagnosis.

	HOR (*n* = 48)			BFS room (*n* = 36)		
	TBCB	TBFB	P value	TBCB	TBFB	P value
Sample size (mm^3^) (median, quartile)	55 (29, 136)	2 (1, 4)	<0.001	70 (29, 144)	6 (4, 8)	<0.001
Pathology yield rate	91.5% (43/47*)	76.6% (36/47*)	0.033	97.2% (35/36)	77.8% (28/36)	0.008
	Lung cancer (*n* = 44)			Benign lesions (*n* = 39)		
	TBCB	TBFB	P value	TBCB	TBFB	P value
Sample size (mm^3^) (median, quartile)	72 (30, 180)	4 (2, 8)	<0.001	42 (24, 76)	2 (1, 6)	<0.001
Pathology yield rate	88.6% (39/44)	75.0% (33/44)	0.054	100% (39/39)	79.5% (31/39)	0.004
	Concentric lesions (*n* = 73)			Eccentric lesions (*n* = 11)		
	TBCB	TBFB	P value	TBCB	TBFB	P value
Sample size (mm^3^) (median, quartile)	60 (30, 144)	4 (2, 8)	<0.001	30 (22, 51)	2 (1.5, 5)	0.006
Pathology yield rate	94.4% (68/72*)	77.7% (56/72*)	0.002	90.9% (10/11)	72.7% (8/11)	0.250

* There was one case lost to follow-up without a final diagnosis.

** HOR: hybrid operating room, BFS: bronchofibroscope, TBCB: transbronchial cryobiopsy, TBFB: transbronchial forceps biopsy.

When stratified by whether guided by AF, significant differences were observed in lesion sizes and equipment used, but not in procedural outcomes. In the HOR group, where AF was used, lesion sizes were significantly smaller [HOR vs. BFS room: 2.4 (1.7, 3.4) mm³ vs. 5.1 (3.5, 6.4) mm³, *p* < 0.001]. The use of thin scopes was higher in the HOR group (13 vs. 9) and ultrathin scopes were exclusively used in the HOR (31 vs. 0). Procedure times were comparable between the HOR and BFS room settings (65.7 ± 24.8 min vs. 63.9 ± 23.5 min, *p* = 0.743). Similarly, total diagnostic yield rates [HOR vs. BFS room: 95.7% vs. 97.2%, *p* = 0.721] and complication rates [HOR vs. BFS room: 10.4% vs. 5.5%, *p* = 0.704] showed no significant differences. TBCB consistently obtained larger samples than TBFB across both settings [TBCB vs. TBFB in HOR: 55 (29, 136) mm³ vs. 2 (1, 4) mm³, *p* < 0.001; TBCB vs. TBFB in BFS room: 70 (29, 144) mm³ vs. 6 (4, 8) mm³, *p* < 0.001]. Furthermore, TBCB had a better diagnostic yield regardless of the procedure location [HOR vs. BFS room: 91.5% vs. 76.6%, *p* = 0.033; 97.2% vs. 77.8%, *p* = 0.008 respectively]. These details are further elaborated in Tables 5 and 6.

**Table 6.** Comparison of patients receiving procedures in the hybrid operation room and bronchoscopic room.

	HOR (*n* = 48)	BFS room (*n* = 36)	P value
Age (mean ± SD)	66.1 ± 12.9	65.8 ± 11.0	0.982
Sex (male)	24 (50%)	25 (69.4%)	0.074
Lung cancer	18 (37.5%)	26 (72.2%)	0.002
Lesion size (cm) (median, quartile)	2.4 (1.7, 3.4)	5.1 (3.5, 6.4)	<0.001
≥2 cm	33 (68.7%)	35 (97.2%)	0.001
<2 cm	15 (31.3%)	1 (2.7%)	
Bronchoscope type			
MP scope	31 (64.6%)	0 (0.0%)	
P scope	13 (27.1%)	9 (25.0%)	
MP scope + P scope	44 (91.7%)	9 (25.0%)	<0.001
Other scope	4 (8.3%)	27 (75.0%)	
Cryoprobe type			
1.1	42 (87.5%)	27 (75.0%)	0.139
Others	6 (12.5%)	9 (25.0%)	
Procedure time	65.7 ± 24.8	63.9 ± 23.5	0.743
Total diagnostic yield	95.7% (45/47*)	97.2% (35/36)	0.721
Complication rate	10.4% (5/48)	5.5% (2/36)	0.704

* There was one case lost to follow-up without a final diagnosis.

** HOR: hybrid operating room, BFS: bronchofibroscope.

## Discussion

This retrospective study demonstrated the benefits of TBCB for diagnosing PPLs. Our findings reveal that TBCB consistently provided a higher pathological yield compared to TBFB, independent of lesion size, use of fluoroscopy or not, or the nature of the pathology (benign or malignant). Nonetheless, TBCB can be performed smoothly whether in a HOR or a BFS room, with only occasional bleeding.

Most previous studies compared the diagnostic effectiveness of TBCB and TBFB, reporting only numerically higher diagnostic yields with TBCB but not statistically significant [22–30]. However, some research indicates that TBCB performs better in more challenging cases, such as with smaller or eccentrically positioned lesions [25,29,31–35]. Notably, Chung et al. [36] suggested that lesions larger than 20 mm and concentric lesions significantly benefit from TBCB, leading to enhanced diagnostic yields. Meanwhile, our data suggests that the advantages of TBCB are universally applicable across different types of lesions.

Chen et al. [35] observed that TBCB is particularly advantageous for identifying benign lesions (80.6% vs. 63.4%, *p* < 0.01), whereas our data suggests the same result (100% vs. 79.5%, *p* = 0.004). Historically, using only TBFB made it more difficult to establish a definitive diagnosis for benign lesions. However, with the introduction of TBCB, microorganisms such as fungi or Acid Fast stain positive bacilli are easier to be identified directly under histological examination. Other benign changes, such as organizing pneumonia, are more easily detected by TBCB as well [35,36]. In our study, by incorporating clinical follow-up, inflammation noted in the initial pathology report was considered a positive diagnostic finding for pneumonia and lung abscess as well. As a result, our diagnostic yield was higher than that previously reported. Moreover, even if we exclude those cases, the diagnostic yield for TBCB is still significantly higher than TBFB (Supplementary Table 1).

Historically, enhancing the diagnostic yield of bronchoscopic biopsies, particularly for small lesions, has posed significant challenges. Various technological advancements have been proposed to address this issue, including the use of ultrathin bronchoscope, AF, VBN, ENB, robotic bronchoscopy, and ROSE [6,37–39]. Nakai et al. [32] and Furuse et al. [40] reported that TBCB, with the aid of VBN, had a better diagnostic yield than TBFB (82.3% vs. 70.8%, *p* = 0.009 and 89.2% vs. 77.6%, *p* < 0.001, respectively). However, these techniques, such as navigation systems and robotic bronchoscopy, are relatively expensive and not widely available. While we didn’t have neither navigation system nor robotic bronchoscopy, we demonstrated that with the combination of ultrathin bronchoscope, manual bronchial tracing, AF, ROSE, and TBCB, the diagnostic yield rate could still be very high (93.8%). Moreover, for lesions that were easier to approach, the diagnostic yield rate was also very high (97.2%) even without AF guidance.

It is widely reported that TBCB can take much bigger specimens than TBFB [20,41]. In our present study, we also showcased that the sample sizes were larger in TBCB than in TBFB, irrespective of lesion size, the use of fluoroscopy, and the benign or malignant nature of the lesion. This capability is particularly critical in the era of precision medicine, where sufficient tissue is required for molecular testing. TBCB has proven to be an effective method for acquiring the necessary samples [42]. In our cohort, every patient (9/9) who opted for next-generation sequencing (NGS) analysis successfully completed the test using samples obtained *via* TBCB. The median tumor cells percentage of these nine specimen was 20% (15%, 40%). Four different panels were used, depending on the primary physician’s decisions, including ACTLung, ACTDrug+, FoundationOne CDx, and Guardant 360. Since NGS analysis is not covered by Taiwan’s National Health Insurance, it limited participation to those who could afford this expensive test and caused low number of patients receiving NGS analysis in our cohort.

Additionally, some of our patients were diagnosed with fungal lung disease solely based on the microorganism morphology in the TBCB sample under histology examination, while they were not found in TBFB specimen and both the microbiology culture of the washing fluid and biopsy tissue yielded negative results. This also revealed the superior diagnostic capability of TBCB over TBFB.

Furthermore, the sequence of TBFB and TBCB remained an issue [41]. In our institution, TBFB was always performed prior to TBCB for following reasons. For infectious disease, microbiology study is required. Taking biopsy tissue for culture with TBFB first can prevent the freezing temperature of TBCB eliminating possible viable microorganisms. Second, despite having r-EBUS to identify and avoid blood vessels at the biopsy site, some of the cases are still more prone to bleeding. Performing TBFB initially helps identify these patients, potentially preventing more severe bleeding that could occur with TBCB. In our study, this approach was reflected in the low incidence of severe complications, with only six cases (7.1%) of grade 3 bleeding and no occurrences of grade 4 bleeding. The incidence of grade 3 bleeding was slightly higher than previous reported (1.2%∼4.8%) [20,33,34,36]. This is likely because we used balloon catheters earlier for precaution in those patients. According to the criteria, the used of balloon catheter automatically classified the bleeding as grade 3.

Interestingly, in our study, two patients diagnosed with adenocarcinoma *via* TBFB showed negative results for malignancy with TBCB (Supplementary Table 2). The first case had a 3.4 cm eccentric lesion located at LB3ai, a region that is not particularly peripheral. Since the lesion was eccentrically located and the bronchus, which was measured 4.9 mm on CT scan, was not narrowed enough to fully encase the 1.1 mm cryoprobe, the tissue obtained *via* TBCB might not be the malignant portion. Additionally, the sample sizes from TBFB and TBCB was 4 mm^3^ and 6 mm^3^ respectively - only a modest difference. However, the biopsy number was 9 for TBFB and 5 for TBCB, which provided TBFB a greater opportunity to capture the cancerous part. In the second case, a 2.2 cm partial solid concentric lesion was located at LB10biiα. Despite the procedure being performed under AF guidance, LB10biiα and LB10biiβ appeared nearly parallel on AF imaging, making it difficult to confirm which bronchial lumen the instruments had entered. During TBFB, the bronchoscope remained fixed in position, and only the biopsy forceps were inserted and withdrawn through the working channel. In contrast, during TBCB, the bronchoscope was removed together with the cryoprobe after each biopsy. It would cause higher chance to misplace the cryoprobe because rEBUS was not used to recheck the lesion between each biopsy. This discrepancy underscores the complementary nature of using both techniques that TBFB could not be totally replaced by TBCB. As suggested by several studies, combining TBFB and TBCB may enhance the overall diagnostic yield, providing a more comprehensive assessment of lesions [27–29,32,33].

Our study had some limitations. Firstly, the relatively small cohort size of 84 patients may limit the statistical power of our findings. Secondly, as a single-institution study, the generalizability of our results to other facilities might be constrained. Despite these limitations, we believe that the insights garnered from our research could be beneficial for other institutions.

In conclusion, TBCB significantly enhances the diagnostic yield for PPLs, irrespective of lesion characteristics. Employing a combination of an ultrathin bronchoscope, AF, ROSE, and TBCB can lead to high diagnostic yields, even in small lesions. Our findings support the use of TBCB as a valuable tool in the diagnostic arsenal for lung pathologies, offering a robust alternative to traditional methods.

## Supplementary Material

Supplementary files clean.docx

## Data Availability

The data that support the findings of this study are available from the corresponding author upon reasonable request.
