# Supplementary material for: Comparative study of transbronchial cryobiopsy and transbronchial biopsy for diagnostic yield in peripheral pulmonary lesions
Source: Ann Med. 2026 Jan 8;58(1):2613456. doi: 10.1080/07853890.2026.2613456 (PMC12784633; doi:10.1080/07853890.2026.2613456)
Supplement: Supplementary files clean.docx [file IANN_A_2613456_SM3218.docx]

Supplementary table 1. Results stratified by diagnostic criterion for benign lesions

|  | Histopathology alone (n=23) | | | Histopathology + clinical diagnosis (n=16) | | |
| --- | --- | --- | --- | --- | --- | --- |
|  | TBCB | TBFB | P value | TBCB | TBFB | P value |
| Pathology yield rate | 100% (23/23) | 65.2% (15/23) | 0.002 | 100% (16/16) | 100% (16/16) | NA |

* TBCB: transbronchial cryobiopsy, TBFB: transbronchial forceps biopsy

Supplementary table 2. Discordant diagnosis pair table

|  | TBCB positive yield | TBCB negative yield | Total |
| --- | --- | --- | --- |
| TBFB positive yield | 62 | 2 | 64 |
| TBFB negative yield | 16 | 3 | 19 |
| Total | 78 | 5 | 83 |

* TBCB: transbronchial cryobiopsy, TBFB: transbronchial forceps biopsy
